# Editorial: Rare immune-mediated diseases- novel insights into underlying mechanisms and therapeutic approaches

**DOI:** 10.3389/fimmu.2023.1273062

**Published:** 2023-08-14

**Authors:** Oded Shamriz, Irit Adini, Yuval Tal

**Affiliations:** ^1^ Allergy and Clinical Immunology Unit, Department of Medicine, Hadassah Medical Organization, Faculty of Medicine, Hebrew University of Jerusalem, Jerusalem, Israel; ^2^ The Lautenberg Center for Immunology and Cancer Research, Institute of Medical Research Israel-Canada, Faculty of Medicine, Hebrew University of Jerusalem, Jerusalem, Israel; ^3^ Department of Surgery, Center for Engineering in Medicine & Surgery, Massachusetts General Hospital, Harvard Medical School, Boston, MA, United States

**Keywords:** pathway, personalized, mechanism, novel, pathophysiology, target

Our understanding of the underlying mechanisms of the immune system has vastly improved over the years. Description of new inborn errors of immunity (IEI) (1), as well as the introduction of novel biological treatments, which target specific pathways in the immune system, have revolutionized the field of clinical immunology.

As a result, treatment of patients with immune-mediated disorders has been increasingly altered from gross immune suppression by corticosteroids and cytotoxic agents to patient-tailored and pathway-specific treatments. For example, patients with lipopolysaccharide (LPS)-responsive and beige-like anchor protein (LRBA) deficiency, who present with immune dysregulation and decrease in CTLA4, are routinely being treated with abatacept, even as a bridge before hematopoietic stem cell transplantation (2). In addition, atopic dermatitis in the context of IEI, such as Wisckot Aldrich syndrome (WAS), and as a manifestation of increased T helper (Th)2-mediated immunity is now considered more manageable with dupilumab, a monoclonal antibody against interleukin (IL)-4/IL-13 receptor α subunit (3, 4). Finally, even the treatment of asthma has been revised. Understanding the different asthma endotypes, whether it is allergic or eosinophilic, will help physicians to offer a personalized treatment by using dupilumab or omalizumab for allergic asthma or anti-IL-5 agents, such as mepolizumab and benralizumab, for the eosinophilic endotype with good clinical outcome (5). This approach allows asthmatic patients to have decreased number of exacerbations without the need of long-period systemic corticosteroids.

Indeed, the treating immunologist must have an in-depth understanding of the underlying immune mechanism of the patient’s disease, to accurately adjust the treatment and to avoid adverse reactions from unnecessary deep immune suppression.

This Research Topic of *Frontiers in Immunology* focuses on novel insights into the mechanisms and innovative treatments of rare immune-mediated diseases. Different immune mechanisms are presented in various studies in this Research Topic. Li et al. analyze type 1 interferon signature in peripheral blood mononuclear cells of patients with idiopathic inflammatory myopathies (IIM). Exploring Th2 immune response, Does et al. depicts in detail the pathophysiology of itch and hypersensitivity reactions of mosquito bites including involvement of mast cells. In addition, Dolitzky et al. describes another important Th2 key player by revealing the regulation of eosinophil activation by apoptotic cells. Lastly, Bader et al. displays a comprehensive analysis of the adaptive immunity against the BNT162b2 mRNA vaccine in adolescents with various immune deficiencies.

There are several studies in this Research Topic that explore the emergence of novel treatments from comprehensive understating of the immune system. Defining eosinophils as key players in drug reaction with eosinophilia and systemic symptoms syndrome (DRESS), Rubin et al. report the use of anti-IL-5 agents as potential treatment with remarkable clinical outcome. Zhao et al. suggest that mesenchymal stem cell-derived extracellular vesicles can be used in multiple sclerosis (MS). Zhen et al. presents an extensive meta-analysis analyzing the use of rituximab in IIM. In addition, Yin et al. reports on the use of transicptome to identify drug repositioning for MS. The authors identify different PI3K-Akt and chemokine signaling pathways as potential novel targets for MS. Another interesting study by Adini et al. presents PR1P, a Vascular Endothelial Growth Factor (VEGF)-stabilizing peptide, as a possible therapeutic target in the inflammatory response of ulcerative colitis.

Finally, Pan et al. reviews the targeting of immune checkpoint inhibitors in anti-neutrophil cytoplasmic antibodies (ANCA)-associated vasculitis (AAV). Different co-inhibitory molecules, such as T cell immunoglobulin (Ig) and mucin domain-containing protein 3 (TIM-3), are suggested by the authors as potential targets for novel biological treatments for AAV.

In conclusion, the accumulating data regarding pathophysiology and novel treatments for immune-mediated disorders are the foundations for the practice of clinical immunologists. Further exploring immune pathways will help develop novel biologics, thus keeping the march towards a personalized and pathway-specific immune treatments.

## Author contributions

OS: Conceptualization, Investigation, Supervision, Writing – original draft. IA: Writing – review & editing. YT: Supervision, Writing – review & editing.
